# Supplementary material for: Splicing Modulators Are Involved in Human Polyglutamine Diversification via Protein Complexes Shuttling between Nucleus and Cytoplasm
Source: Int J Mol Sci. 2023 Jun 1;24(11):9622. doi: 10.3390/ijms24119622 (PMC10253306; doi:10.3390/ijms24119622)
Supplement: Supplementary file 1 [file ijms-24-09622-s001.zip › Supl_Caption_Ledgends.docx]

Table S1: Name of genes coding proteins that interact with both PolyQ-binding proteins and PolyQ-disease-causing proteins.

Table S2: Gene Ontology of co-interactors between polyQ-binding proteins and polyQ-containing proteins.

Figure S1. PPI networks among two polyQ-binding proteins (PQBP1 and VCP) and five polyQ-disease causing proteins (HTT, AR, ATXN1, ATXN3, and ATXN7) using HIPPIE. (a) The PPIs network among the five proteins listed in Table 1 (a) were input as queries of HIPPIE network option with “score filter” set as 0.83. Other options were not changed as default setting. (b) The network obtained by the same except brain regions were selected as “tissue filter.”

Figure S2. Integrated annotation of ID regions and SCOP domains obtained through D^2^P^2^.

Results of each protein was shown as follows:

ATXN1: https://d2p2.pro/view/sequence/up/P54253

AR: https://d2p2.pro/view/sequence/up/P10275

NCOA3: https://d2p2.pro/view/sequence/up/Q9Y6Q9

EP300: https://d2p2.pro/view/sequence/up/Q09472

CREBBP: https://d2p2.pro/view/sequence/up/Q92793

HTT: https://d2p2.pro/view/sequence/up/P42858

USP7: https://d2p2.pro/view/sequence/up/Q93009

RNABP9: https://d2p2.pro/view/sequence/up/Q96S59

PQBP1: https://d2p2.pro/view/sequence/up/O60828

VCP: https://d2p2.pro/view/sequence/up/P55072
