# Supplementary figures and images for: Splicing Modulators Are Involved in Human Polyglutamine Diversification via Protein Complexes Shuttling between Nucleus and Cytoplasm
Source: Int J Mol Sci. 2023 Jun 1;24(11):9622. doi: 10.3390/ijms24119622 (PMC10253306; doi:10.3390/ijms24119622)

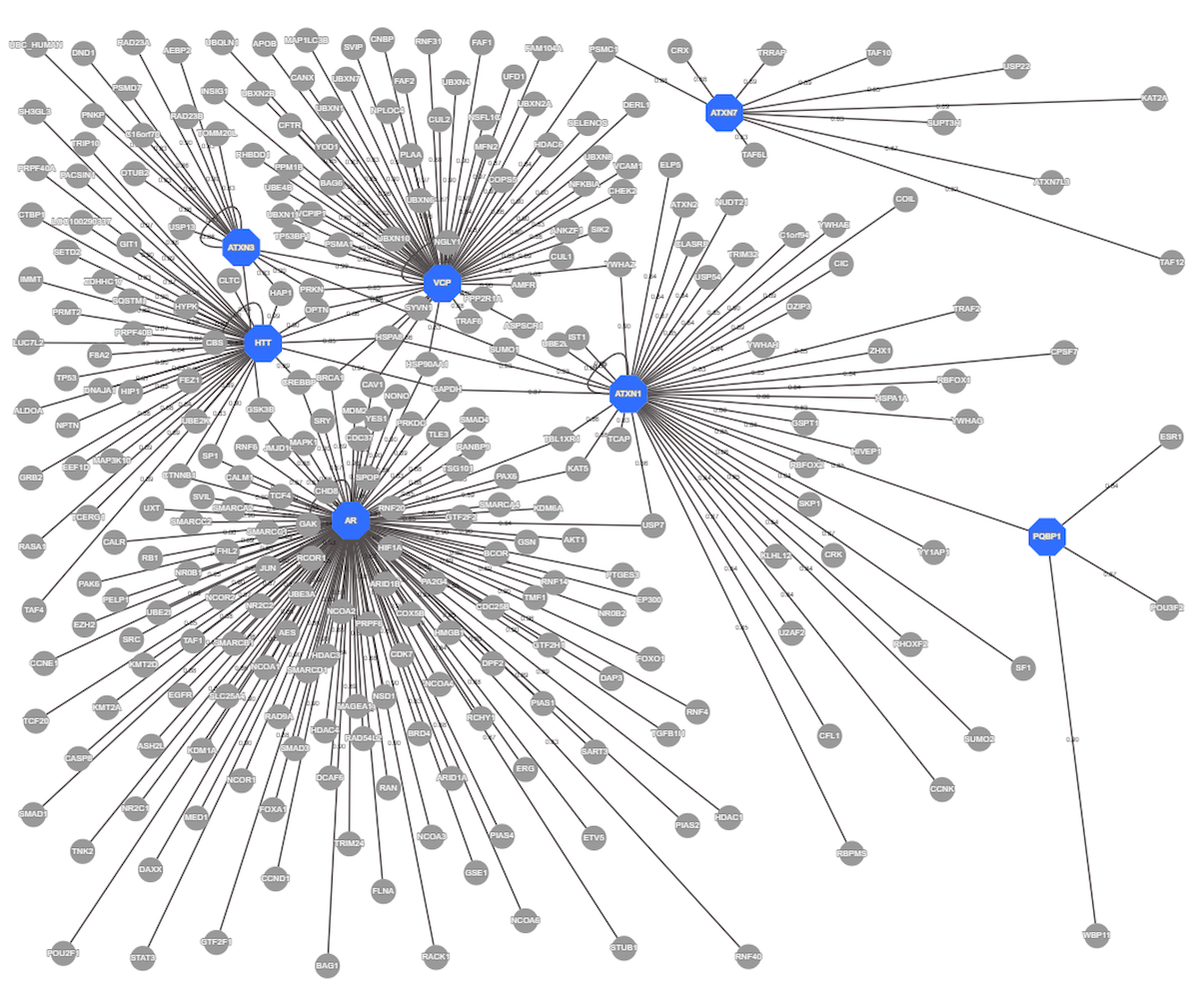

Supplement: Supplementary file 1 [file ijms-24-09622-s001.zip › FigS1a_subnetwork7protein20230405HIPPIE_ScrnshotAdptive.png]

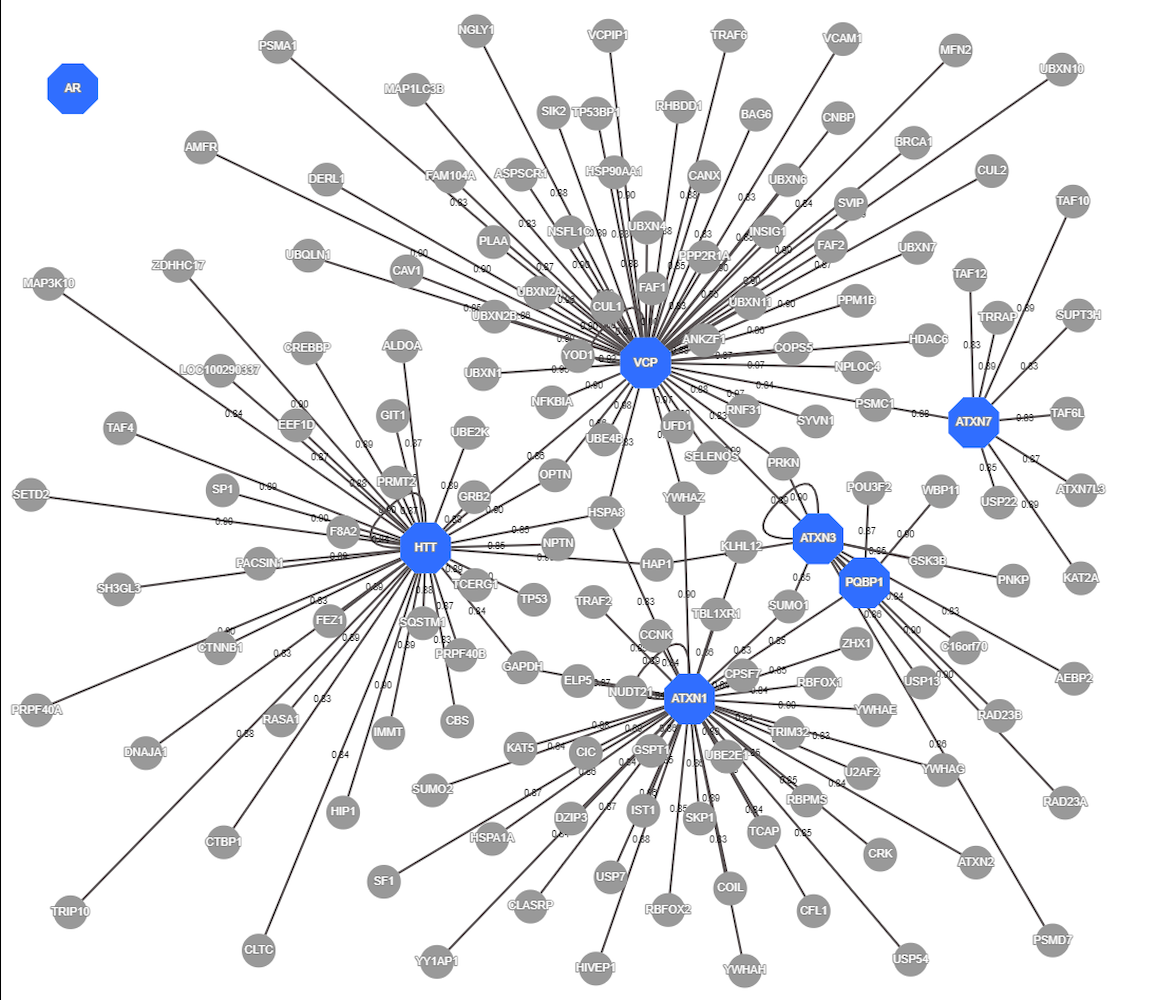

Supplement: Supplementary file 1 [file ijms-24-09622-s001.zip › FigS1b_subnetwork7protein_Brain20230405HIPPIR_ScrnshotAdaptive.png]
